# Profile of asymmetrical retinopathy of prematurity in twins

**DOI:** 10.4103/0301-4738.62645

**Published:** 2010

**Authors:** Rajvardhan Azad, Parijat Chandra, Sourabh D Patwardhan, Aparna Gupta

**Affiliations:** Dr. Rajendra Prasad Centre for Ophthalmic Sciences, All India Institute of Medical Sciences, New Delhi - 110 029, India

**Keywords:** Retinopathy of prematurity, screening retinopathy of prematurity, twins

## Abstract

**Background:**

In twin births, both babies have the same gestational age and pre-natal conditions. However, twins may develop a varied retinopathy of prematurity (ROP) course depending on birth weight and other systemic factors.

**Objective:**

To study the profile of asymmetric ROP in twins

**Design:**

Retrospective study

**Setting:**

Tertiary ROP referral eye hospital.

**Materials and Methods:**

The profile of 56 pairs of twins with ROP were studied and analyzed for differences in zone or need for treatment, while studying possible causes for the varied outcome.

**Results:**

In 45 pairs of twins (80%) the disease progressed identically in both eyes, while in 11 pairs (20%) the ROP showed differences in zone or need for treatment. Four of these pairs were discordant. In 3 of these 4 pairs, the heavier birth weight twin had a more severe ROP course.

**Conclusions:**

Twins can present with asymmetric ROP course, and it is therefore essential to examine both twins as per screening protocols.

Retinopathy of Prematurity (ROP) is a potentially blinding vasoproliferative disease in premature babies. The presentation and course of ROP is determined by a complex interaction of several risk factors like gestational age (GA), birth weight (BW) and many systemic risk factors like anemia, sepsis, jaundice, and multiple blood transfusions.[1] In cases of twin births, since both babies have the same GA and are exposed to the same pre-natal conditions; these babies might present a varied ROP disease course depending on various factors like BW and other systemic diseases they develop.[2,3] Thus, a study model with premature twin babies would help us assess, better, the role of other risk factors altering the course of ROP. This study aims to analyze the profile of asymmetric ROP in twins.

## Materials and Methods

We retrospectively analyzed the data of 56 pairs of twins with ROP diagnosed and treated at our tertiary ROP referral center in the last three years. We analyzed the profile and risk factors of these twin pairs, and studied the final disease outcome, in terms of regression (spontaneous or after treatment) or progression. These twins were analyzed for differences in zones or need for treatment, and possible causes for the varied outcome were analyzed. The twin pair was considered asymmetrical if there was a difference of one zone or two stages in the worst eyes. The babies were considered discordant when there was a difference in birth weight of 15%.

All babies were screened and treated as per standard ROP guidelines.[4] Zone and stage mentioned in Table 1 indicate most severe zone or stage reached during follow-up or presentation.

**Table 1:** Profile of twins with asymmetric retinopathy of prematurity presentation and course

Twin pair number	Gest. age (wks)	Birth weight (grams)	Discordancy in weight	Risk factors	Right eye-zone	Right eye-stage	Right eye-plus	Left eye-zone	Left eye-stage	Left eye-plus	Laser/ cryo	Results
1	A 31	1625	+	O, A, J, Asp	2	2		2	2			Regressed
	B	1300	+	O,J	1	4b		1	4b		Yes	R-stage 5
												L regressed
2	A 30	1300		J	1	3	+++	1	3	+++	Yes	Regressed
	B	1200		J	2	3		2	3		Yes	Regressed
3	A 30	1220		O, S, Asp, BT	1	2	+++	1	2	+++	Yes	Regressed
	B	1130		O, J, BT	2	2	+	2	2	+	Yes	Regressed
4	A 28	1250	+	O	1	5		1	5			Untreatable
	B	850	+		2	3	++	2	3	++	Yes	Regressed
5	A 32	1700	+	S, J, RDS	2	3	+	2	3	+	Yes	Regressed
	B	1250	+	S	3	2		3	1			Regressed
6	A 29	1100			2	3	++	2	3	++	Yes	Regressed
	B	1000			1	3	+++	1	3	+++	Yes	Regressed
7	A 28	1100			2	3		2	3		Yes	Regressed
	B	1100			3	1		3	1			Regressed
8	A 30	1600		O	2	2		2	2			Regressed
	B	1450			3	1		3	1			Regressed
9	A 29	1260		O, BT	2	5		2	4b		Yes	Progressed
												to stage 5
	B	1200		O, BT	3	1		3	1			Regressed
10	A 28	1250	+		1	5		1	5			Untreatable
	B	1000	+		2	3	++	2	3	++	Yes	Regressed
11	A 28	1240		O	2	2		2	2			Regressed
	B	1135		O, S, J, RDS	1	2	++	1	2	++	Yes	Regressed

O-Excess oxygen; A-Apneic episodes; J-Jaundice, ASP-Asphyxia; BT-Blood transfusion; S-Sepsis, RDS-Respiratory distress syndrome; PDA-Patent ductus arteriosus

## Results

A data analysis of 56 pairs of ROP affected twins showed the average GA was 29 ± 2.2 weeks and average BW was 1236 ± 319 grams. In 45 pairs of twins (80%), the disease progressed identically in both eyes with respect to zone, need for treatment, regression and progression. Of these 45 pairs of twins with symmetric ROP, five pairs had zone 1 disease in both eyes, which underwent laser and regressed; 17 pairs had zone 2 ROP in both eyes, of which nine were treated with laser and one pair with cryotherapy; the remaining seven pairs received no intervention and regressed spontaneously. Of the treated pairs, eight regressed and two progressed to higher stages. Nineteen pairs had zone 3 ROP, which regressed without any intervention. Four pairs had inoperable stage 5 disease in both eyes.

Of these 56 pairs, 11 pairs of twins (20%) had a difference of zone or two stages [Table 1]. The babies were discordant in four pairs. The heavier twin had a more severe form of ROP in three of these pairs. On further analysis, some of the babies who progressed were exposed to additional risk factors like sepsis, blood transfusion, jaundice, respiratory distress syndrome, excess of oxygen, and apnea episodes [Table 1]. There was also no significant difference in GA and BW between the babies of the pairs who had less severe form (GA of 29.3 ± 1.3 and BW of 1297 ± 188gm) and those who had more severe form (GA 29.3 ± 1.3 and BW of 1239 ± 206gm). [*P* = 1.000 for GA, *P* = 0.390 for BW].

## Discussion

It is well known that post-natal factors considerably influence ROP development and progression.[1] It is agreed that ROP screening in VLBW (very low birth weight) twins may be conducted according to the same standard protocols as for singletons.[2] Twins provide a good study model since they have the same GA and are exposed to the same prenatal risk factors. Thus, it helps us to analyze the role of birth weight and systemic complications on progression of ROP in two premature babies.

Usually there is no significant difference in stage of ROP between infants of single-gestation pregnancies vs. those of multiple-gestation pregnancies.[3] We observed that in 45 pairs the ROP progressed similarly with respect to zone and stage of disease.

Our study results suggest that the twins had variable courses of ROP in 11 of the 56 pairs (20%). This is useful information because it suggests that if ROP has a mild presentation and course in one twin, it is not necessary that the other twin will follow the same course; and 20% of these twins may differ in their presentation and progression of ROP. Thus, there is a need to examine and follow-up both babies regularly as per screening guidelines. This is especially important in developing countries where uninformed parents believe that if ROP is regressing in one twin, they think the other twin will regress too and fail to bring the other twin for screening.

Another notable finding is that in three of the four discordant pairs, the heavier birth weight baby presented with a more severe ROP course than the lower birth weight baby. This is important since it is usually expected that the smaller birth weight baby would develop a more severe course of ROP. In a related study, Fellows *et al*. studied discordant twins and reported that 38% (10 sets) of the lower birth weight infants had higher grades of ROP than their twin, while 23% (six sets) of the heavier birth weight twins had higher grades of ROP than their smaller siblings.[5] Though some additional risk factors have been identified, their significance could not be analyzed due to multiple variables and it would need another study for the same. These facts can also be extrapolated to triplets, quadruplets, and pentuplets.[6]

This article reinforces the fact that screening of all babies is necessary in cases of multiple births, and birth weight alone cannot be relied upon as a single factor to predict the severity and course of ROP, as even heavier siblings can develop severe ROP and may present with variable course. This study model may be further applied to non-twins, which might help us understand the X-factor, which governs the severity of ROP in the postnatal period.
